# Microvascular Plug Embolization of Anterior Spinal Artery Bearing Segmental Arteries Prior Aortic Stenting: Technique and Safety

**DOI:** 10.1007/s00270-024-03909-4

**Published:** 2024-11-27

**Authors:** Sinan Deniz, Osman Öcal, Matthias Fabritius, Daniel Puhr-Westerheide, Gizem Abaci, Moritz Wildgruber, Muzaffer Reha Ümütlü, Mustafa Gök, Jan Stana, Barbara Rantner, Nikolaos Tsilimparis, Jens Ricke, Max Seidensticker

**Affiliations:** 1 Department of Radiology, University Hospital, Ludwig Maximilian University of Munich, Munich, Germany; 2 Department of Diagnostic and Interventional Radiology, Heidelberg University, Heidelberg, Germany; 3 Department of Radiology, Adnan Menderes University, Aydin, Türkiye; 4 Sydney Medical School and School of Health Sciences, Faculty of Medicine and Health, University of Sydney, Sydney, NSW Australia; 5 Department of Vascular Surgery, University Hospital, Ludwig Maximilian University of Munich, Munich, Germany

**Keywords:** Angiography, Anterior spinal artery, Coils, Embolization, Microvascular plug, Segmental artery, Spinal cord ischemia

## Abstract

**Purpose:**

This study aims to present our experience with superselective embolization of the anterior spinal artery-bearing segmental artery (ASAbSA) using a microvascular plug (MVP) during the minimally invasive segmental artery coil embolization (MISACE) procedure prior endovascular repair of the thoracoabdominal aortic aneurysms.

**Methods:**

We retrospectively evaluated all MISACE procedures performed between May 2018 and July 2023, where MVP was deployed into an angiographically confirmed ASAbSA. Data were analyzed regarding interventional details, technical aspects, and safety protocols. The standard procedure for MVP embolization involves detaching the plug after 10 min, provided no neurological symptoms occur.

**Results:**

A total of 22 patients underwent MVP deployment into the proximal segmental artery supplying the ASAbSA. There were no instances of non-target embolization or segmental artery dissection. Furthermore, none of the patients experienced temporary or permanent spinal cord ischemia.

**Conclusion:**

MVP deployment into the ASAbSA is a safe strategy for protecting the spinal cord during preemptive embolization of segmental arteries prior to endovascular aortic repair.

**Graphical Abstract:**

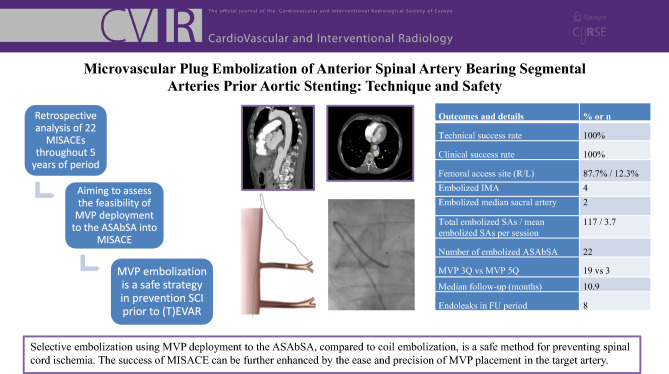

## Introduction

Ischemic spinal cord injury (SCI) leading to paraplegia is a well-recognized and devastating complication of both open and endovascular aortic aneurysm repair [1, 2].

Griepp’s collateral network (CN) theory [3] has led to strategies designed to enhance collateral blood flow to the spinal cord, either through staged procedures [4] or by preconditioning the CN using minimally invasive segmental artery coil embolization (MISACE) [5].

The goal is to stimulate the development of collateral pathways supplying the anterior spinal artery (ASA), preserving retrograde blood flow before performing the (T) EVAR procedure.

However, segmental artery embolization poses a risk of ischemic spinal cord complications through two mechanisms: an acute reduction in blood flow to the spinal cord and the potential for inaccurate coiling, which can inadvertently obstruct the ASA. In such cases, removing the deployed coils becomes impractical. To address this, we use a modified approach involving a microvascular plug (MVP) for the segmental artery. The MVP offers more precise deployment, and importantly, can be temporarily positioned and retrieved if SCI symptoms emerge.

The objective of this retrospective study was to assess the safety and clinical outcomes of this technique.

## Material and Methods

### Patients

Study approval was obtained from an institutional review board. All patients had a signed written procedural informed consent, a study-specific informed consent was waived due to its retrospective nature. All patients who underwent MISACE procedure between May 2018 and July 2023 are evaluated, and the procedures with an angiographic-proven ASA have been included. Patient demographics as well as technical aspects such as puncture side, used catheters, relevant segmental artery to the ASA, maximal diameter of the aortic aneurysm, levels and numbers of occluded segmental arteries or any additional visceral branches, complications, and related follow-up data were assessed throughout the electronic patient recording system of our tertiary care center. The etiologies and the Crawford classification of the pathologies were analyzed [2].

### MISACE Procedure

**Fig. 1:**
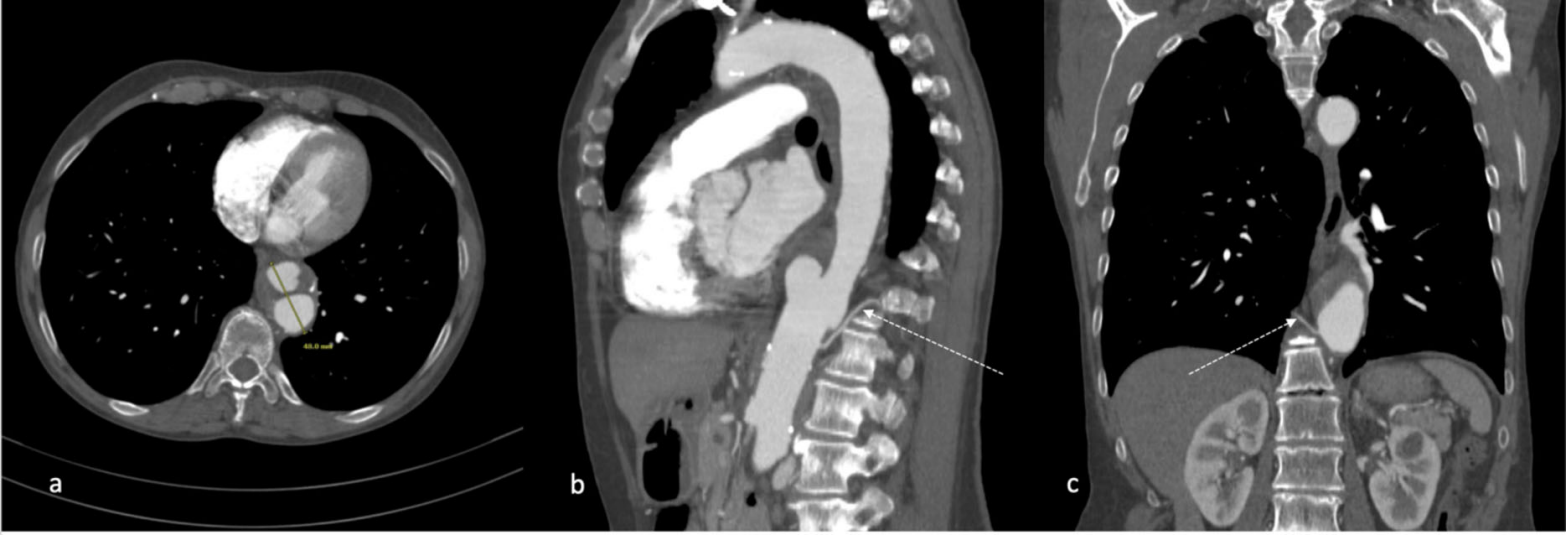
CTA of the aorta. **a** Axial diameter of the aneurysm. **b** Sagittal reconstruction with an avid L-1 segmental artery **c** Coronal projection with a right Th-12 segmental artery. *CTA: computed tomography angiography, L: lumbal, Th: thoracal

The number and anatomy of the segmental arteries (SAs) relevant to preemptive embolization were evaluated using the patient’s preexisting CTA (computed tomography angiography) scans. The CTA protocol included an ECG-triggered arterial phase CT scan of the thoracoabdominal aorta, performed after the injection of 100 ml of intravenous contrast media. This was followed by 3D reconstructions of the acquired data (Fig. 1).

**Fig. 2:**
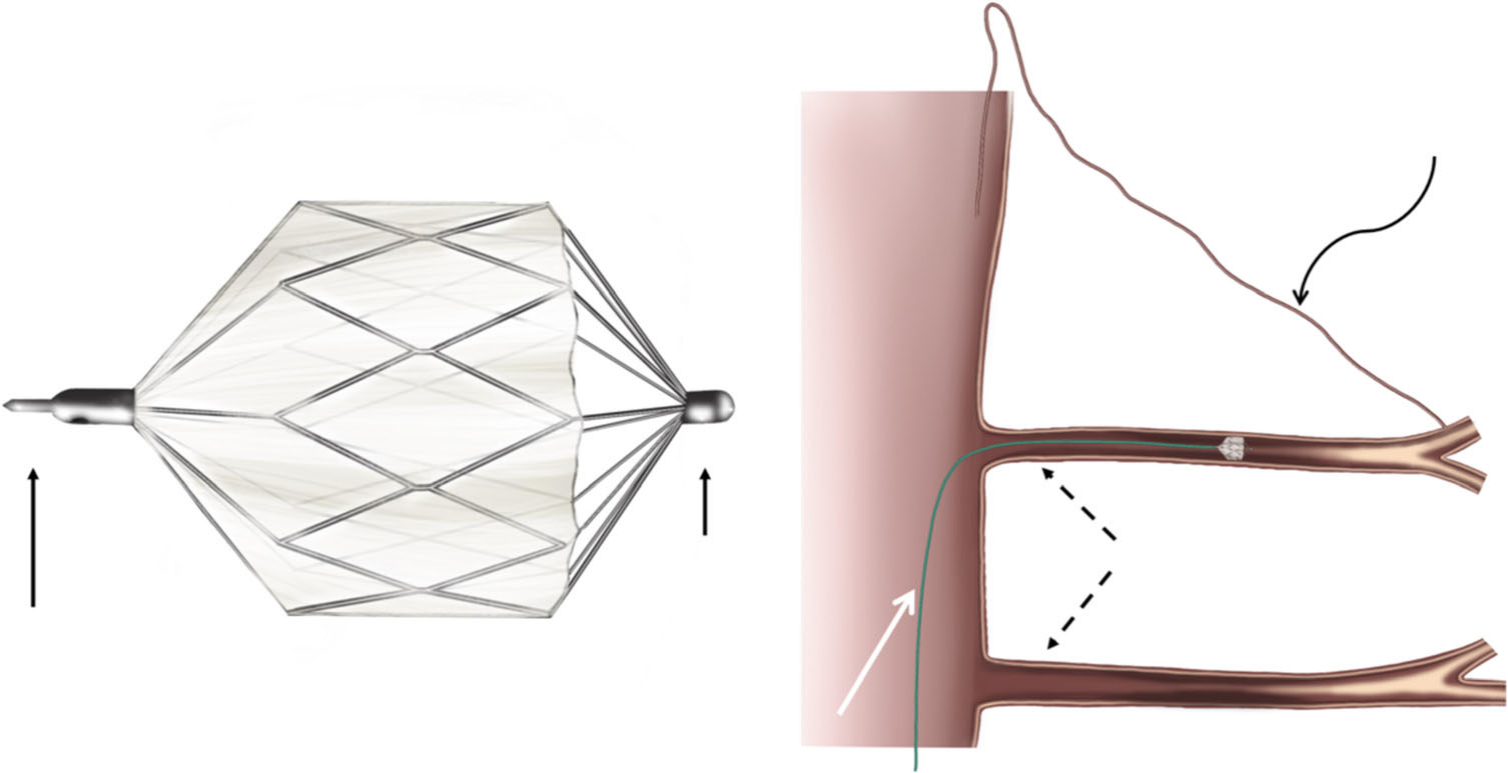
Schematic drawings. **a** of a microvascular plug (MVP), with the longer arrow pointing out the proximal marker, the short arrow being the distal marker. **b** Representation of a MVP placement in a segmental artery (SA: dotted black arrows) with a distally originated anterior spinal artery (ASA: curved black arrow) with the white arrow pointing the wire inside of a microcatheter with a placed but yet not permanently deployed MVP. *MVP: microvascular plug, SA: segmental artery, ASA: anterior spinal artery

After standart angiographic access through the groin, diagnostic catheters, such as Sim-I, C2, JR1, H1, and Special (Cook®, Angiodynamics®, Terumo®) were used to access the planned segmental arteries. A microcatheter of 2,4 F or 2,7 F of size (Progreat/Terumo®) with inner diameters of 0.021″ for MVP-3Q and 0.027″ for MVP-5Q, Medtronic®) was utilized to access the segmental arteries superselectively (Fig. 2).

Following the catheterization, the presence of the ASA was excluded with contrast injections, and the SA was occluded proximally with coils. The goal was to occlude up to four pairs of segmental arteries in a single session. If necessary, additional sessions were performed to complete the preemptive embolization.

### MVP Deployment

Once the anterior spinal artery-bearing segmental artery (ASAbSA) was angiographically suspected, it was carefully identified using focused and detailed angiograms. A microvascular plug (MVP) 3Q (for target vessels 1.5 to 3 mm) or 5Q (for target vessels 3 to 5 mm) was then precisely delivered through the microcatheter. After the intravenous administration of 5000 IU of heparin, the plug was carefully deployed in a very proximal position, with the aim of preserving retrograde perfusion to the ASA.

**Fig. 3:**
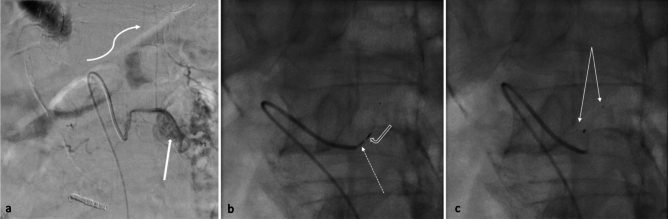
Stepwise deployment of the MVP at the anterior spinal artery bearing segmental artery (ASAbSA), proximal to the offspring of the ASA. **a** DSA of the L1-left lumbal artery feeding the anterior spinal artery (ASA, white solenoid arrow), straight white arrow pointing the offspring of the ASA. **b** Optional temporarily embolization with a proximally placed MVP-3Q Plug: Dotted white arrow shows the tip of the microcatheter, curved empty arrow points out the screw/detachment point of the plug. **c** Deployed plug at the proximal segmental artery (SA), with proximal and distal radiopaque markers pointed out with white arrows. *MVP: Microvascular plug, ASAbSA: Anterior spinal artery bearing segmental artery, ASA: anterior spinal artery, DSA: digital subtraction angiography, SA: segmental artery

Before permanently detaching the device, we waited for 10 min with the MVP fully deployed but not yet released, in order to monitor for any immediate neurological side effects (e.g., paresthesia, paraparesis). This was done by continuously assessing the patient’s sensory responses and motor function, particularly in the feet. Once no adverse effects were observed, the MVP was permanently detached from its wire using a counterclockwise rotation (Fig. 3c).

Technical success was defined as the precise deployment and permanent release of the microvascular plug at the intended location following a successful test occlusion. Clinical success was defined as the absence of any clinical signs of spinal cord ischemia.

### Follow-up

All patients were closely monitored and neurologically assessed as inpatients for four days following the procedure, with no temporary or permanent neurological deficits indicating spinal cord ischemia (SCI) observed throughout the entire follow-up period. Patient data were reviewed for morbidity, mortality, and complications during follow-up, extending up to six months after TEVAR. Complications were evaluated using the CIRSE Classification System.

### Statistical Analysis

Statistical analysis was performed using SPSS Statistics 23.0 (IBM, New York). Continuous variables were presented as mean and standard deviation, whereas categorical parameters were presented as number and percentage. Univariable analysis of the relationship between procedural characteristics and technical (or clinical) success was assessed using Chi square and t-Tests. A two-sided-*p*-value < 0.05 was considered statistically significant.

## Results

A total of 22 patients (18 men, 4 women, mean age 64.2 ± 10.8 years) treated across 32 sessions were identified. 117 segmental arteries, four mesenteric inferior arteries, and two median sacral arteries were coil-embolized, besides 22 occluded ASAbSA via MVPs. Six patients underwent two sessions of MISACE, while two patients had three sessions. All sessions were technically successful. Sixteen patients had TAAAs according to Crawford classification (Table 1.), whereas six patients had isolated AAAs; which were para- or suprarenal aneurysms with the need of a f-EVAR with according high proximal landing zones.

**Table 1:** Baseline characteristics of the study population

Baseline characteristics	Number
Age (mean ± standard deviation)	64.2 ± 10.8
Gender (male)	19 (82.6%)
AAA	6
TAAA Crawford I Crawford II Crawford III Crawford IV Crawford V	16 4 7 - 4 1
Mean AAA diameter in mm	62.1 ± 8.9

Angiography-related minor complications were seen in 4 (18.1%) patients (Table 2). One patient had contrast media allergy during the procedure, which was treated with IV antihistamine. One patient had a self-limiting groin hematoma, and two patients had groin pseudoaneurysms, which were treated with percutaneous thrombin injection.

**Table 2:** Pre- and periprocedural details

Outcomes and technical details	% or n
Technical success rate	100%
Success rate	100%
Femoral access site (right/left)	87.75%/12.25%
Embolized IMA	4
Embolized median sacral artery	2
Total embolized SAs/mean embolized SAs per session	117 / 3.7
Number of embolized ASAbSA	22
MVP 3Q vs MVP 5Q	19 vs 3
CIRSE complication classification system Grade I Grade II Grade III and above Neurologic complications	4 2 1 0
Median follow-up (months)	10.9 (3d–49 m 11d)
Endoleaks in FU period Type Ia Type Ib Type II Type III Type Ia and III combined	8 1 2 1 3 1

There were no cases of non-target embolization or misplacement of MVPs. Two patients experienced a temporary sensory dysfunction on their lower extremities during contrast injection into the segmental artery before plug deployment. One of the patients developed sudden back pain on the fourth postprocedural day and underwent an emergent CT scan, which revealed a rupture of the aortic aneurysm. He has been directly taken into the OR but could not survive the incident.

The remaining 21 patients underwent (T) EVAR in a median period of 44.5 days (range, 3–115 days). During the median follow-up of 10.9 months, no cases of temporary or permanent neurological deficits were reported.

At the one-month follow-up, eight cases had endoleaks (Table 2). Of these, two resolved by the six-month follow-up, while the remaining six patients received additional treatment based on the type of leakage: four patients underwent graft and/or chimney extensions, and two received percutaneous embolization with nonalcoholic liquid embolic material (Onyx, Medtronic®).

## Discussion

SCI is a devastating complication after aortic repair of both open and endovascular techniques [6]. Etz et al. demonstrated that the SC perfusion is not dependent on only one artery but also has a potentially underdeveloped collateral system [7], which can be beneficial if segmental arteries (SAs) are occluded after a (T)EVAR procedure.

The anterior spinal artery (ASA), primarily supplied by the vertebral arteries, receives significant blood flow from the Adamkiewicz artery. However, this is not the sole source of spinal cord perfusion. Instead, the spinal cord is nourished by an axial network of small arteries, including not only segmental but also some pelvic and mesenteric arteries [8]. It has been shown occlusion of a SA triggers an ischemic stimulus, provoking subsequent angiogenesis [9], which can trigger the increase in small arterial vessel diameters and facilitate the resauration of spinal cord perfusion through alternative arterial pathways [7, 10]. As a result, staged repair of the aneurysmal sac compared to single session significantly reduces paraplegia rates following extensive thoracoabdominal aortic aneurysm repairs [4].

MISACE is an alternative approach to staged repair, which aims to restore and provoke the collateral network of the spinal cord blood supply and also help to prevent the backflow to the aneurysmal sac through the segmental arteries. However, coil embolization of the segmental artery bearing Adamkiewicz artery carries the risk of acute spinal cord ischemia, especially if the ASA offspring is affected by embolization.

In our cohort, we deployed a microvascular plug to this segmental artery without detaching it (Fig. 3b), and waited for 10 min to foresee any potential neurologic deficits. This technique resulted in no cases of temporary or permanent spinal cord ischemia. This can be explained by the unique feature of microplugs and their precise and retrievable placement through very low profile microcatheters.

In a 2022 study by Addas et al., 2 out of 14 patients who underwent MISACE with coil embolization developed paraparesis postoperatively [11]. MVPs have gained a wide use because of their steerability in smaller vessels and their quicker occlusive properties compared with coil-embolization [12].

In our cohort, aside from brief sensory dysfunction in two cases, none of the patients developed paraparesis or paraplegia. This temporary phenomenon could speculatively be caused from the bubbly contrast injection and/or from the potential vasospasm caused by prior wire manipulations, which were very self-limiting and lasted for a couple of moments, not causing an obstacle to a controlled MVP deployment.

The occurrence of an aneurysm rupture on the 4th post-interventional day, while the patient was awaiting planned surgery, prompts a reconsideration of the optimal timing between intervention and surgery. Although there is no clear consensus on this issue, the mean intervention-to-surgery interval in our cohort was 44.5 days. The patient in question had been diagnosed with a 59-mm aneurysm 7 months prior. It remains unclear whether the rupture was spontaneous or related to the intervention. Brown et al. reported that the annual rupture risk for aortic aneurysms larger than 6 cm in diameter is 14.1%, compared to a 1% risk for aneurysms in the 50–59 mm range [13]. Although evalution of the optimum sequencing of MISACE and following TEVAR was not part of our analysis, this fact needs to be considered during the risk–benefit evaluation of the procedure.

Our study has several limitations. It is a retrospective analysis with a relatively small patient sample and lacks a control group. Additionally, there was no standardized monitoring for potential neurological adverse effects caused by spinal cord ischemia. Despite these limitations, there was no selection bias, as all patients who underwent the MISACE procedure were reviewed and included in this study.

## Conclusion

Preemptive selective embolization of the segmental arteries aims to stimulate the remodeling of the collateral network. Our results demonstrate that selective embolization using MVP deployment to the ASAbSA, is a safe method for preventing spinal cord ischemia. The success of MISACE can be further enhanced by the ease and precision of MVP placement in the target artery.
